# Improving the Catalytic Performance of BaMn_0.7_Cu_0.3_O_3_ Perovskite for CO Oxidation in Simulated Cars Exhaust Conditions by Partial Substitution of Ba

**DOI:** 10.3390/molecules29051056

**Published:** 2024-02-28

**Authors:** Nawel Ghezali, Álvaro Díaz Verde, María José Illán Gómez

**Affiliations:** MCMA Group, Inorganic Chemistry Department, Materials Institute of the University of Alicante (IUMA), Faculty of Sciences, University of Alicante, 03690 Alicante, Spain; gn11@alu.ua.es (N.G.); alvaro.diaz@ua.es (Á.D.V.)

**Keywords:** Ba/Cu/Mn perovskite-type mixed oxides, sol–gel synthesis, CO oxidation, Ce/La/Mg

## Abstract

The sol–gel method, adapted to aqueous media, was used for the synthesis of BaMn_0.7_Cu_0.3_O_3_ (BMC) and Ba_0.9_A_0.1_Mn_0.7_Cu_0.3_O_3_ (BMC-A, A = Ce, La or Mg) perovskite-type mixed oxides. These samples were fully characterized by ICP-OES, XRD, XPS, H_2_-TPR, BET, and O_2_–TPD and, subsequently, they were evaluated as catalysts for CO oxidation under different conditions simulating that found in cars exhaust. The characterization results show that after the partial replacement of Ba by A metal in BMC perovskite: (i) a fraction of the polytype structure was converted to the hexagonal BaMnO_3_ perovskite structure, (ii) A metal used as dopant was incorporated into the lattice of the perovskite, (iii) oxygen vacancies existed on the surface of samples, and iv) Mn(IV) and Mn(III) coexisted on the surface and in the bulk, with Mn(IV) being the main oxidation state on the surface. In the three reactant atmospheres used, all samples catalysed the CO to CO_2_ oxidation reaction, showing better performances after the addition of A metal and for reactant mixtures with low CO/O_2_ ratios. BMC-Ce was the most active catalyst because it combined the highest reducibility and oxygen mobility, the presence of copper and of oxygen vacancies on the surface, the contribution of the Ce(IV)/Ce(III) redox pair, and a high proportion of surface and bulk Mn(IV). At 200 °C and in the 0.1% CO + 10% O_2_ reactant gas mixture, the CO conversion using BMC-Ce was very similar to the achieved with a 1% Pt/Al_2_O_3_ (Pt-Al) reference catalyst.

## 1. Introduction

Currently, one of the most urgent challenges of the society is the control of pollutants evolved by car engines that cause several environmental and health problems [1,2]. Among other issues, the development of cheap, efficient, and stable catalysts for the control of the CO levels in the exhaust of automobile engines is a key topic to which the scientific community is dedicating huge efforts. As an example, the denoted “Target 150 °C” was established, with the objective of achieving a 90% reduction of hydrocarbon (HC), CO, and NO_x_ emissions from automobile engines at 150 °C [3]. Thus, focusing on CO removal, it is well known that catalysts based on noble metals (mainly Pt, Pd, and Rh) are highly efficient, but they present two main drawbacks, as they are expensive [4] and suffer an inhibition by CO adsorption that hinders the dissociative chemisorption of oxygen, which is required for CO oxidation [5]. In this line, mixed oxides with perovskite-type structure (ABO_3_) [6,7,8,9,10,11,12,13,14,15] are considered as an interesting family of catalysts for achieving this target.

Perovskites are mixed oxides with a general formula ABO_3_, in which *A* represents a lanthanide, alkali, or alkaline earth metal (presenting either +2 or +3 oxidation states), and *B* is a transition metal with +4 or +3 oxidation states. Assuming a cubic unit cell, *A* cations show a 12th coordination index, while the coordination index of *B* cations is 6 [16,17,18,19]. Perovskites turn out to be very interesting catalysts since the catalytic properties can be tailored by modifying the composition without a significant distortion of the structure. The changes could include, among others, a partial substitution of *A* and/or *B* cations, and/or the modification of the stoichiometry of *A* and/or *B* cations. After these modifications, the electroneutrality of the solid could be lost and, to recover it, two compensation mechanisms have been proposed [20]: (i) the so-called ionic compensation mechanism, which involves tailoring the amount of a *A* or *B* cations, and (ii) the denoted electronic compensation mechanism that implies a change in the oxidation state of *A* or *B* cations. These charge compensation mechanisms modify the properties of the perovskites and, consequently, their catalytic performance.

In a previous paper of the authors [13], in which a series of Ba_0.9_A_0.1_Cu_0.3_Mn_0.7_O_3_ (A = Mg, Ce, La, Ca, Sr) perovskite-type mixed oxides were used for soot oxidation under simulated GDI exhaust conditions, it was concluded that the partially substituted samples presented a higher selectivity to CO_2_ than the BaCu_0.3_Mn_0.7_O_3_ raw sample, so it is expected that they potentially catalyse the CO to CO_2_ oxidation. Additionally, in a recently published article [21], it was proven that the catalytic performance of BaMnO_3_ perovskites for CO oxidation reaction is improved by the partial substitution of Ba cation by Ce, La or Mg. Consequently, a useful strategy to further enhance the catalytic performance for CO oxidation of Ba_0.9_A_0.1_Cu_0.3_Mn_0.7_O_3_ would be the partial replacement of the Ba cation.

Thus, in this paper, a series of Ba_0.9_A_0.1_Cu_0.3_Mn_0.7_O_3_ (A = Ce, La, Mg) perovskites was prepared, characterized, and tested for the CO oxidation in different conditions simulating the composition of the cars exhaust. The conditions employed for the CO oxidation reaction are those previously used for the Ba_0.9_A_0.1_MnO_3_ (A = Ce, La, Mg) samples [21], which are: (i) 1% CO and 1% O_2_ in He, as an approximation to the gaseous mixture in the exhaust of a gasoline car engine; (ii) 1% CO and 10% O_2_ in He, for analysing the effect of using a higher oxygen concentration with respect to (i) conditions; and (iii) 0.1% CO and 10% O_2_ in He, for simulating the CO oxidation in a very large excess of oxygen, which could be close to the CO/O_2_ ratio in the actual Diesel Oxidation Catalytic (DOC) devices or in the exhaust of oxy-fuel engines (a large excess of O_2_ and very low amount of CO) [22].

## 2. Results and Discussion

### 2.1. Characterization

Table 1 displays the nomenclature, the BET surface area (obtained from N_2_ adsorption data), some relevant XRD data (such as the average crystallite size calculated by applying the Williamson-Hall method [23]), and the Cu and A (Ce, La, or Mg) weight percentages (determined by ICP-OES).

The ICP-OES data indicate that the mixed oxides contain the amounts of metals (Cu and Ce, La, or Mg) that were added during the sol–gel synthesis. After doping with A- metal, the BET surface area increased from 3 m^2^/g to 6 for BMC and to 7 m^2^/g for BMC-Ce and BMC-La, respectively. The very low surface areas are consistent with the expected for solids with a very low porosity development, as mixed oxides with perovskite structure are [6,16,24], and they were likely due to the relatively high calcination temperature used (850 °C) [25,26], as this temperature determines the ultimate physical and chemical properties of solids.

**Figure 1:**
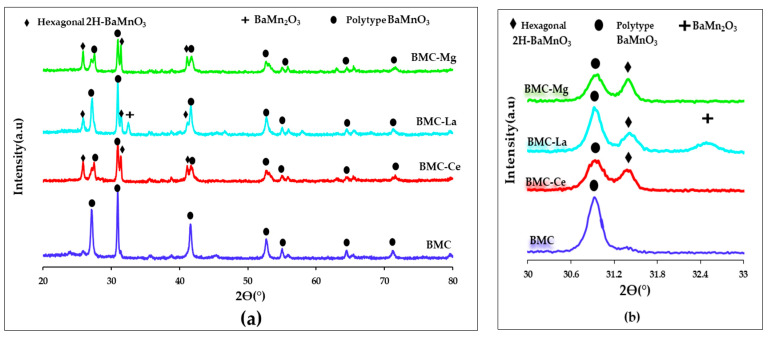
(**a**) XRD profiles and (**b**) magnification of the 2Θ diffraction angle area corresponding to hexagonal and polytype BaMnO_3_ main diffraction peaks.

The XRD patterns of BMC and BMC-A mixed oxides are displayed in Figure 1, being the most relevant related data included in Table 1. All samples presented crystal phases corresponding to a perovskite structure:

(i) For the BMC sample, the BaMnO_3_ polytype structure was the main crystal phase. This structure is a modification of the original hexagonal perovskite structure (shown for BaMnO_3_ (BM) [27]), which is formed due to the partial substitution of Mn by Cu (in the B site of the perovskite lattice), which leads to a different rearrangement of the MO_6_ octahedra [28].
(ii) For BMC-A, the addition of Ce, La or Mg provoked a partial reversion of the polytype structure to the hexagonal 2H-BaMnO_3_ structure (PDF number: 026-0168, denoted by the ICDD, the International Centre of Diffraction Data), so the two crystal phases coexisted in the BMC-A samples. This fact could be considered as evidence of the effective insertion of the A metal into the perovskite lattice. Additionally, it is noteworthy that, for the BMC-La sample, a diffraction peak corresponding to BaMn_2_O_3_ (PDF number 073-0997, denoted by the ICDD, the International Centre of Diffraction Data) was discernible as a minority crystal phase.

The coexistence of the hexagonal and polytype perovskite structures seems to indicate that the presence of A metal hinders the incorporation of copper into the perovskite lattice. However, as copper is not identified as CuO, it should be present either as an amorphous phase (so it cannot be observed by XRD), or it should be inside the perovskite network but generate a low degree of distortion of the original hexagonal perovskite structure due to the presence of A metal. In this line, the coexistence of both hexagonal and polytype structures was previously observed for Ba_0.9_Mn_0.7_Cu_0.3_O_3_ and Ba_0.8_Mn_0.7_Cu_0.3_O_3_ perovskites [29]. To clearly display the changes of the main XRD peaks corresponding to the polytype crystal structure, in Figure 1b, the range of 2θ diffraction angle from 30.5° to 32.5° is amplified. Thus, even though the maximum of the main diffraction peak of the polytype structure is not modified (located at a 2θ of c.a. 30.9°), a decrease in the intensity is featured, showing BMC-Mg the lowest intensity and BMC-La the highest one (see data in Table 1). This modification has to be related to the location of A metal into the perovskite structure [30], which, in turn, depends on the ionic radii of A cation [31], that is, 146.4 pm for Ba(II), 53.0/73.0 pm for Mn(III)/Mn(IV), 65.0 pm for Cu(II), 105.2/90.6 pm for Ce(III)/Ce(IV), 107.3 pm for La(III), and 65.0 pm for Mg(II). Based on these data, it is suggested that:

(i) Ce and La cations should be placed in the A site of the perovskite structure because their ionic radii are close to the Ba(II) radius.
(ii) The Mg cation should be inserted into the B site of the perovskite structure (so, partially replacing Cu and/or Mn) since the ionic radius of Mg(II) is closer to the ionic radius of Mn(III) and of Cu(II) than to the radius of Ba(II). In fact, in a previous article [30], it was also concluded that Mg(II) partially replaces Ti(IV) instead Ba(II) in the B position of the BaTiO_3_ perovskite structure.

Note that the insertion of A metal (either in *A* or *B* position of the perovskite structure) also affected the average crystal size and the lattice strain, determined using the Williamson–Hall method [23] and included in Table 1. Both the average crystal size and the lattice strain exhibited lower values than for the raw BMC perovskite. Thus, if A metal were present in the catalytic formulation, the crystal growth of the polytype phase seemed to be hindered, and the lattice strain decreased; as it could be expected based on the size of A cations (see above), the nature of A metal was relevant, with BMC-La presenting the lowest values and BMC-Mg the highest one from BMC-A samples. This is because La(III) presented the highest ionic radius and Mg(II) presented the lowest one, so they generated the highest and the lowest degree of distortion in the polytype structure of BMC, respectively. Conversely, *a* and *c* lattice parameters of the polytype structure were not appreciably affected by the presence of A metal, except for the BMC-Ce sample, which showed a smooth distortion of the polytype structure as *a* parameter slightly decreased (5.6 Å for BMC-Ce versus 5.8 Å for BMC).

In summary, the addition of A metal in BMC perovskites caused the coexistence of the hexagonal and the polytype crystal phases, as well as the decrease in the average crystal size and in the lattice strain of the polytype structure, and did not significantly affect the cell parameters. However, the degree of these modifications depends on the nature of the A metal: BMC-Mg presented the highest proportion of the hexagonal structure (as deduced from the intensity of the main XRD peaks of the polytype and of the hexagonal structures), BMC-La presented the lowest average crystal size and lattice strain, and BMC-Ce showed a slight change in the cell parameters of the polytype structure.

**Figure 2:**
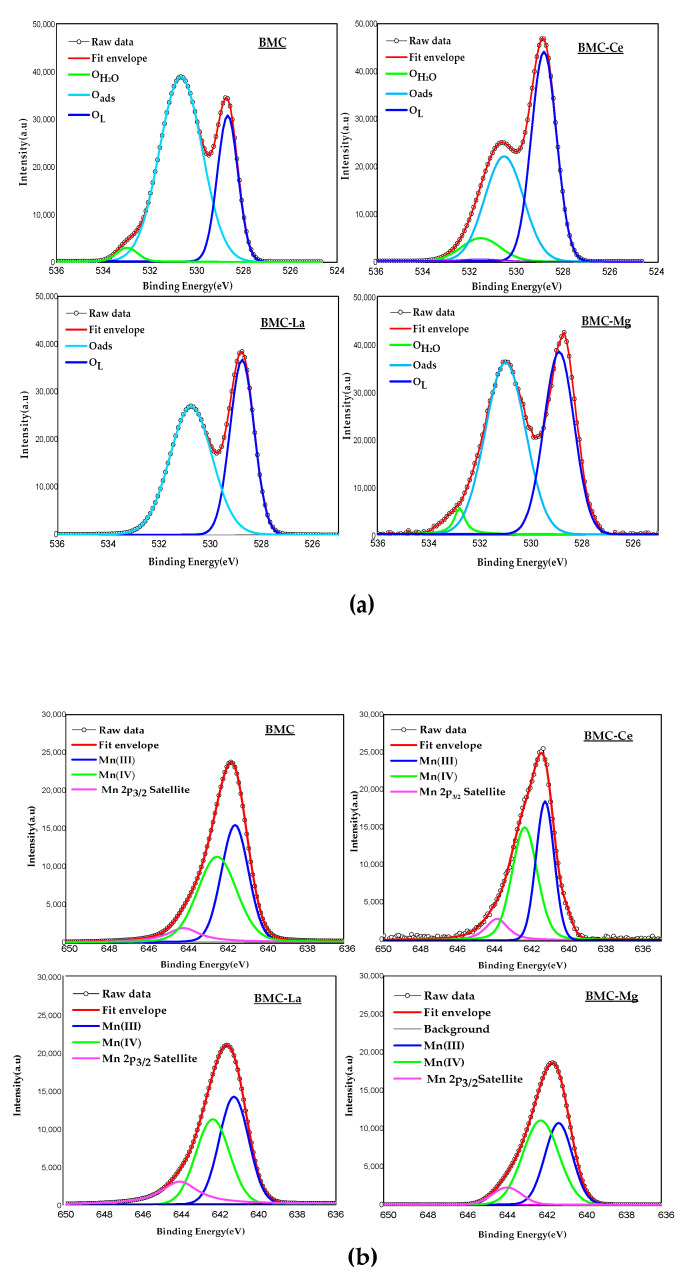

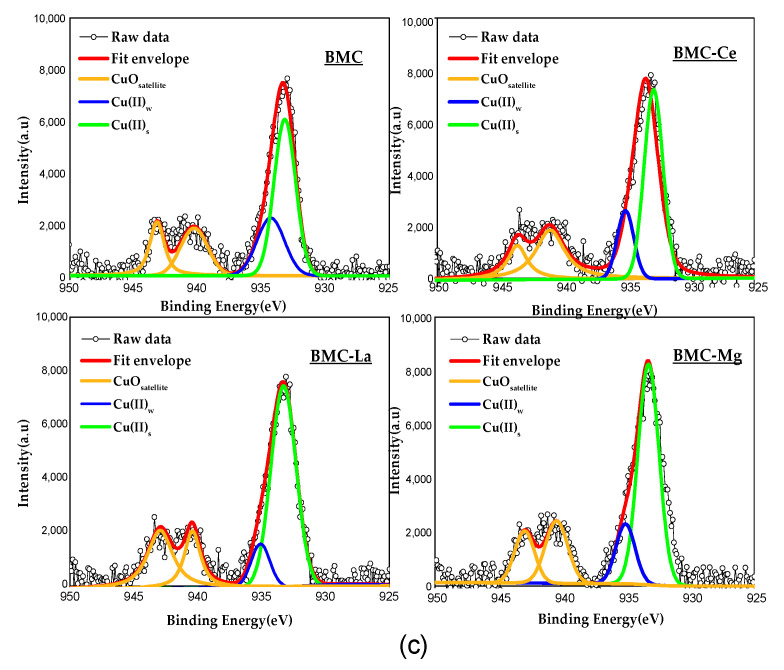
XPS spectra in the (**a**) O 1s, (**b**) Mn 2p_3/2_, and (**c**) Cu 2p_3/2_ core-level regions.

The surface composition of samples was analysed using XPS technique, with XPS spectra of O 1s, Mn 2p_3/2_, and Cu 2p_3/2_ for the core-level regions illustrated in Figure 2; in Table 2, the most significant XPS data are compiled.

In the O 1s spectrum shown in Figure 2a, the three peaks previously described for perovskites [32,33,34] and attributed to chemisorbed water species (O_H2O_), adsorbed oxygen species (named “O_ads_” that include surface carbonate, hydroxyl groups, peroxide, and superoxide ions, and defect sites with low oxygen coordination [32]) and lattice oxygen (O_L_) were detected for the BMC, BMC-Ce, BMC-La, and BMC-Mg samples. The binding energies corresponding to the maximum of the O_L_ and O_ads_ peaks are featured in Table 2, where a slight chemical shift towards higher binding energies (maximum shift of 0.2 eV for BMC-Mg) was detected. It is important to consider that a shift of the band towards lower binding energies implies the presence of a richer electronic environment, whereas a shift towards higher binding energies indicates the opposite. Thus, as a result of the loss of oxygen from the MnO_6_ octahedra (which takes place for achieving the electroneutrality of positive and negative charges due to the presence of Mn(III) (see below) and Cu(II) [35]), the O_L_ peak was slightly displaced to higher binding energies for BMC-Mg (0.2 eV), as Mg(II) partially filled the Mn sites, and for the BMC-Ce sample (0.1 eV) because Ce(IV)/Ce(III) partially replaces Ba(II). Note that, as the O_L_/(Ba + Mn + Cu + A) ratios were lower than the theoretical value for ABO_3_ perovskite (1.5), oxygen vacancies (defect sites with low oxygen coordination) were present on the surface of all samples, as could be expected due to the imbalance of positive and negative charges. Note also that, after the introduction of A -metal, a slight increase of this ratio with respect to BMC was detected.

In the Mn 2p_3/2_ XPS spectra (Figure 2b), three peaks were identified with maxima at ca 641.0 eV, 642.0 eV, and 644.0 eV, that can be attributed to the Mn(III), Mn(IV), and Mn(III) satellite peak, respectively [36,37]. It was observed that only the introduction of Mg(II) affected the binding energy of the Mn(III) and Mn(IV) peaks which, indeed, seems to be associated with the different location of Mg(II) in the Mn site instead in the Ba site [30]. Concerning the Mn(IV)/Mn(III) ratio, a significant modification was not detected after A metal doping and, as these ratios were higher than 1, it was concluded that Mn(IV) was the main oxidation state on the surface.

Finally, in the Cu(II) 2p_3/2_ spectra (Figure 2c), three peaks at binding energies of ca. 933.0, 934.5, and 940.0–943.0 eV were identified, corresponding to Cu(II) with strong (Cu(II)_s_) and weak (Cu(II)_w_) interaction with the perovskite surface for the two formers, and to Cu(II) satellite peak [38] for the latest peak. Because of A metal doping, an increase in the binding energy corresponding to the maximum of Cu(II)_w_ was detected, revealing the presence of a poorer electronic environment than in raw BMC, which was more relevant for BMC-La, probably due to the most intense distortion of the structure found in this sample (see XRD data). Focusing the attention on the Cu/(Ba + Mn + Cu + A) ratios, they were lower than the theoretical value (0.15) for all the samples, indicating that Cu(II) was partially incorporated into the perovskite lattice. However, the Cu/(Ba + Mn + Cu + A) ratio depends on the A metal: it did not change for BMC-Mg, a slight increase was found for BMC-La, and it decreased from 0.09 to 0.07 for BMC-Ce. So, it seems that the degree of the partial insertion of Cu into the lattice depends on the A metal, presenting BMC-Ce the highest degree of copper inserted into the lattice of BMC.

**Figure 3:**
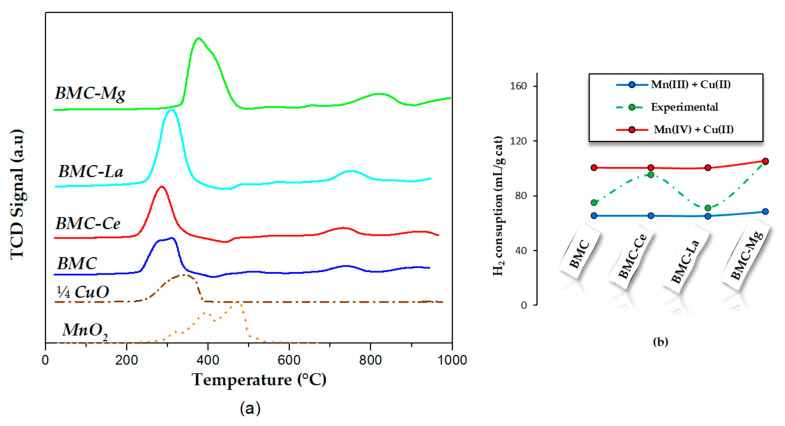
(**a**) H_2_–TPR profiles and (**b**) H_2_ consumption (mL/g of catalyst).

H_2_–TPR experiments were performed to evaluate the reducibility of samples, with the profiles shown in Figure 3a, where those corresponding to MnO_2_ and CuO (with the intensity divided by 4 to be comparable with the other profiles) used as references were also included. The H_2_-TPR profile for MnO_2_ showed two closely overlapping reduction peaks with maxima at about 400 °C and 500 °C [39] that corresponded to different reduction processes: the first peak to the reduction of MnO_2_ or Mn_2_O_3_ to Mn_3_O_4_, and the second one was associated with the reduction of Mn_3_O_4_ to MnO.

In the H_2_-TPR profile of perovskites, three reduction peaks were identified:

(i) At the lowest temperature (200 °C–400 °C), the sharp peak corresponded to the reduction of Cu(II) to Cu(0) but also includes the reduction of Mn(IV)/Mn(III) to Mn(II). As these peaks were located at temperatures lower than those observed in CuO and MnO_2_ references, a synergetic effect has to exist between Cu and Mn [37].
(ii) At intermediate temperatures (700 °C–800 °C), the low-intensity peak was assigned to the desorption/reduction of oxygen species.
(iii) At the highest temperatures (900 °C–1000 °C), the very low-intensity peak was related to the reduction of bulk Mn(III) to Mn(II).

Focusing the attention on the temperature for the maximum of the sharp reduction peak, it seems that the presence of A metal affected the reduction of Mn(IV)/Mn(III) to Mn(II) and of Cu(II) to Cu(0):

- The introduction of Ce provoked a shift towards lower temperatures (285 °C), so Mn and Cu were more easily reduced than in BMC (309 °C), probably due to the contribution of the Ce(IV)/Ce(III) redox pair.
- After the introduction of Mg, an increase in the reduction temperature was observed (376 °C), so, the reduction of Mn and Cu seems to be more difficult. It could be associated to the different location of Mg(II) in the perovskite lattice (described above), which seemed to decrease the Mn-Cu synergetic effect.

On the other hand, using the profiles depicted in Figure 3a, the experimental hydrogen consumption was estimated, and it is compared in Figure 3b with the theoretical hydrogen consumption, calculated considering the total reduction of manganese and copper in two scenarios: (i) manganese as Mn(III) and copper as Cu(II), represented by the blue line; and (ii) manganese as Mn(IV) and copper as Cu(II), represented by the red line. It is noteworthy that, for BMC-Mg and BMC-Ce, the experimental values aligned more closely with the assumption of Mn(IV) + Cu(II), while for BMC and BMC-La, they were closer to Mn(III) + Cu(II). This observation suggests that, for BMC-Ce and BM-Mg, Mn(IV) was the main oxidation state in the bulk, meanwhile, for BMC and for BMC-La, the main species was Mn(III). Note that XPS indicates the coexistence of both Mn(III) and Mn(IV) oxidation states on the surface, but Mn(IV) was present in a higher proportion for all samples.

**Figure 4:**
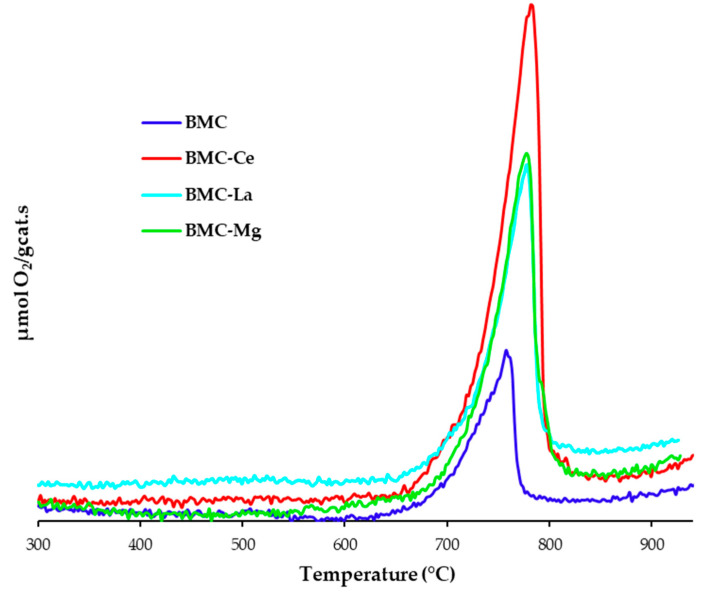
O_2_–TPD profiles.

To end the discussion of the characterization results, the influence of A metal on the O_2_–TPD profiles under He flow is depicted in Figure 4. Note that only one defined peak at temperatures higher than 700 °C was detected, which was assigned to the desorption of oxygen from the perovskite lattice (β-O_2_) [6,16]. This oxygen was generated by the oxidation of O^2−^ in the lattice, which was coupled with the reduction of Mn(IV) to Mn(III), of Cu(II) to Cu(I) [40,41,42], and for BMC-Ce, also to Ce(IV) to Ce(III) reduction [43]. After A metal doping, the temperature of the maximum appears at higher values (781 °C versus 757 °C for BMC), with this shift being directly related to the energy of the Mn-O and Cu-O bonds, which seemed to be modified by the presence of A metal through the modification of the Mn-Cu synergetic effect. On the other hand, the total amount of β-O_2_ released was estimated by calculating the area under the peak between 700 °C and 900 °C, being (in μmol/g sample) 113 for BMC-Ce, 86 for BMC-Mg, 60 for BMC-La, and 49 for BMC. So, the addition of A metal increased the amount of β-O_2_ released and, consequently, the oxygen mobility, which achieved the highest value for BMC-Ce due to the contribution of the Ce(IV)/Ce(III) redox pair.

In summary, the characterization data reveal that:

(i) The presence of A metal favours the hexagonal structure versus the polytype structure (formed in BMC sample due to the distortion caused by the Cu(II) insertion into the lattice), allowing the coexistence of the two crystal phases.
(ii) Mn(IV) and Mn(III) coexisted on the surface of all samples, with Mn(IV) in a higher proportion. However, in the bulk, the main oxidation state depended on the A-metal: Mn(IV) was the main one for BMC-Ce and BMC-Mg, while Mn(III) was for BMC and BMC-La.
(iii) All samples featured surface oxygen vacancies.
(iv) The partial substitution of Ba(II) in BMC led to an enhancement of the reducibility and of the lattice oxygen mobility, mainly for BMC-Ce due to the contribution of the Ce(IV)/Ce(III) redox pair.

### 2.2. Catalytic Activity

**Figure 5:**
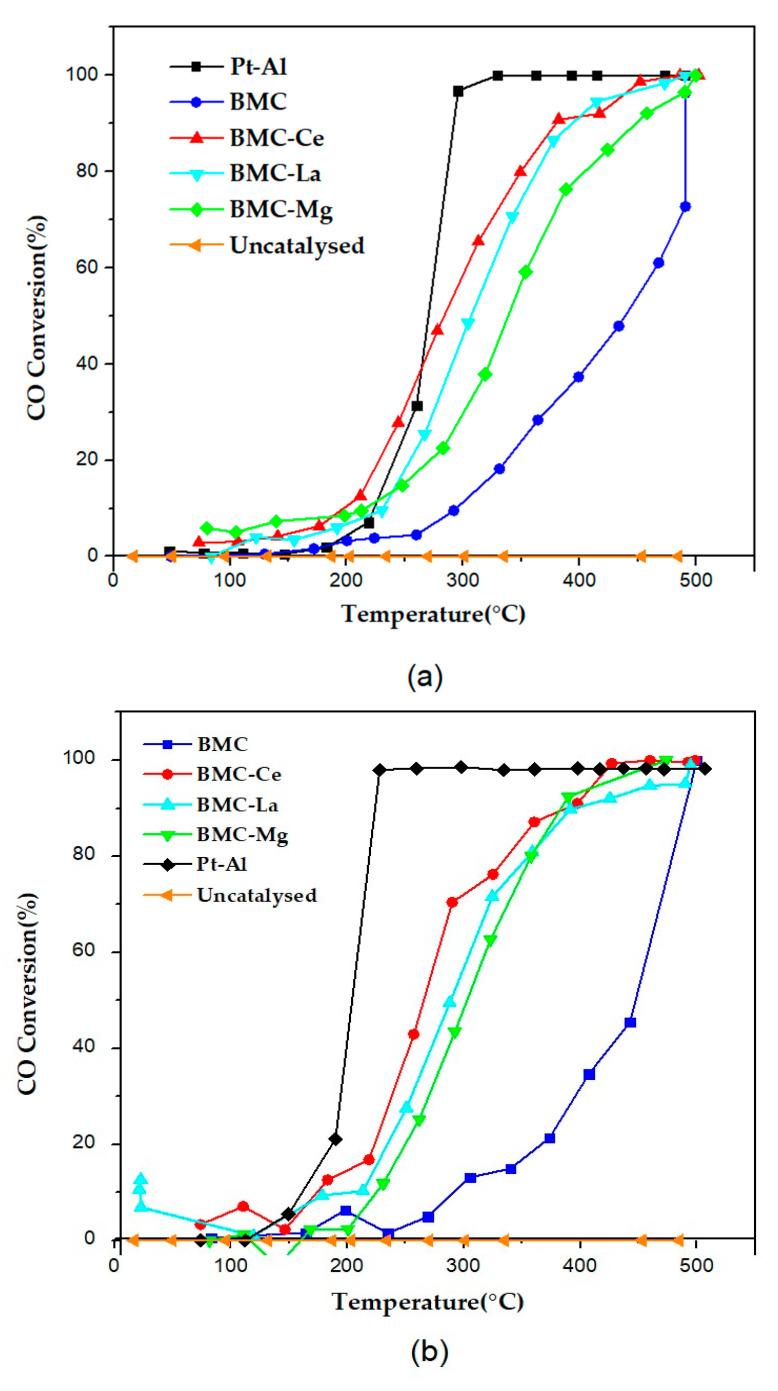

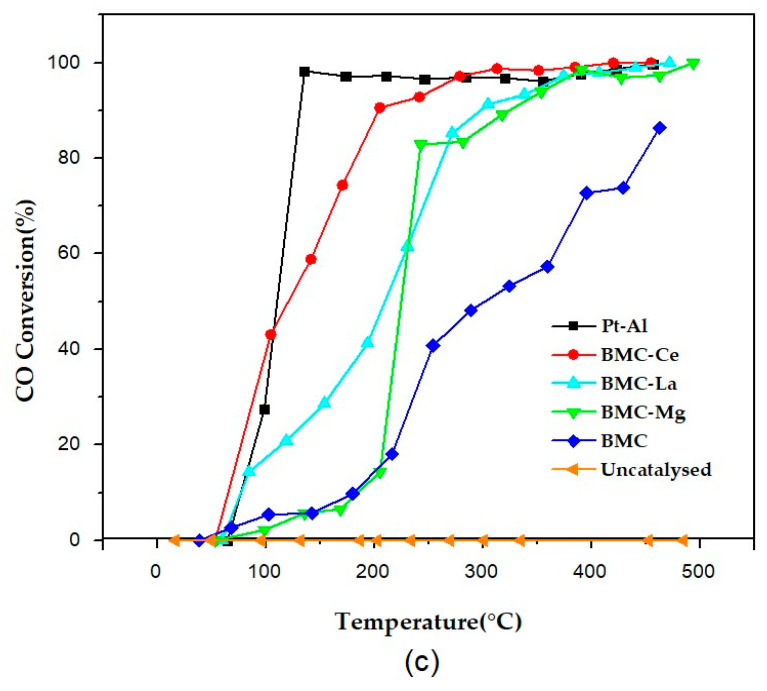
CO-TPR conversion profiles in (**a**) 1% CO + 1% O_2_, (**b**) 1% CO + 10% O_2_, and (**c**) 0.1% CO + 10% O_2_.

In Figure 5, the CO conversion profiles for the BMC and BMC-A samples under the three atmospheres tested (1% CO + 1% O_2_, 1% CO + 10% O_2_, and 0.1% CO + 10% O_2_) are shown, including the profile of a 1% Pt/Al_2_O_3_ commercial catalyst (Pt-Al, from Sigma-Aldrich) used as reference. As the reaction did not take place in the absence of a catalyst (see “Uncatalysed” profile), all samples increased the percentage of CO conversion in the range of temperatures tested and for the three gas mixtures used, even though all perovskites featured lower CO conversion percentages than the achieved using the Pt-Al reference. Note that all the profiles of BMC-A were shifted towards lower temperatures with respect to BMC, so the addition of A metal increased the conversion of CO at T < 500 °C, as it was previously observed for the BM-A series [21]. Additionally, as it could be expected due to the catalytic role of copper [44,45], and as it was previously concluded for BM and BMC raw perovskites [27]; for BMC-A samples, the percentage of CO conversion achieved at a certain temperature was higher than the featured for BM-A samples [21], showing a higher improvement if oxygen was in excess in the reactant mixture. This enhancement seems to be related to the contribution of copper, which offers additional active sites for the adsorption/activation of CO and O_2_ [46]. Consequently, the presence of A metal in BMC decreased the T_50%_ values (temperature to achieve the 50% of CO conversion) that are compiled in Table 3. BMC-Ce was the most active catalyst as it presented the lowest T_50%_ (so, the highest drop with respect to BMC), which was similar to that of Pt-Al. This suggests that the coexistence of surface copper species, oxygen vacancies, a high proportion of bulk and surface Mn(IV), and the contribution of the Ce(IV)/Ce(III) redox pair allowed BMC-Ce to achieve the highest improvement in the catalytic performance of BMC for CO oxidation. In fact, the catalytic role of Ce in the CO oxidation reaction is well established [45,47,48,49], and it was also previously detected for BM-A series [21], even though BM-Ce was not the best catalyst. So, it seems that, in the presence of copper, the role of Ce is boosted.

By comparing the T_50%_ values for the three reactant mixtures tested, it is evident that, according to literature [50,51] and to our previous results for BM-A series [21], there was a clear effect of the CO/O_2_ ratio in the catalytic performance. Thus, in an excess of oxygen (1% CO + 10% O_2_ and 0.1% CO + 10% O_2_ gas mixtures), the T_50%_ decreased for almost all samples (see data compiled in Table 3), with this effect being more relevant as the excess of oxygen increased. Thus, as concluded for the BM-A series [21], the reactant gas mixture in which the perovskite-based catalysts seem to be more effective for improving the CO conversion was 0.1% CO + 10% O_2_, so, in the presence of the largest excess of oxygen. Additionally, the effect of A metal depended on the CO/O_2_ ratio; in 1% CO + 10% O_2,_ the increase in the CO conversion was similar for BMC-Ce, BMC-La, and BMC-Mg, but, for the reactant mixture with the lowest CO/O_2_ ratio (0.1% CO + 10% O_2_), BMC-Ce featured the best performance.

Based on the literature, it can be assumed that the CO oxidation reaction using perovskites as catalysts follows the Langmuir–Hinshelwood (LH) mechanism, which involves the adsorption of CO and O_2_ molecules followed by the reaction between them (being the adsorption of both species the rate-limiting step) [50,51,52,53]. It has been also established that, if a reactant mixture with a low CO percentage is used (like 1% CO + 10% O_2_ or 0.1% CO + 10% O_2_), oxygen will be preferentially chemisorbed on the surface oxygen vacancies (as it is in excess) and, subsequently, the CO molecules will be adsorbed on the remaining free active sites [54,55]. On the other hand, Royer et al. [11] suggested that, if a strong CO inhibition is detected, it means that the two gases compete for the same adsorption sites. For the BMC-A series, the T_50%_ value (Table 3) increased if the CO percentage was also increased from 0.1% to 1%, so CO and O_2_ competed for the active sites, as it was previously concluded for BM-A series [21]. However, for 1% CO in the reactant mixture, the increase in the O_2_ content from 1% to 10% did not affect to T_50%_, as it was previously concluded for BM-A series [21], because Cu provides additional active sites for CO and O_2_ activation.

**Figure 6:**
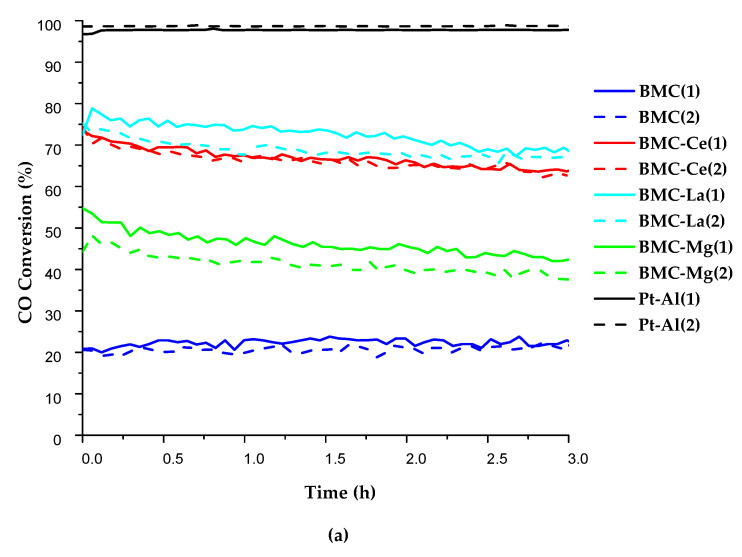

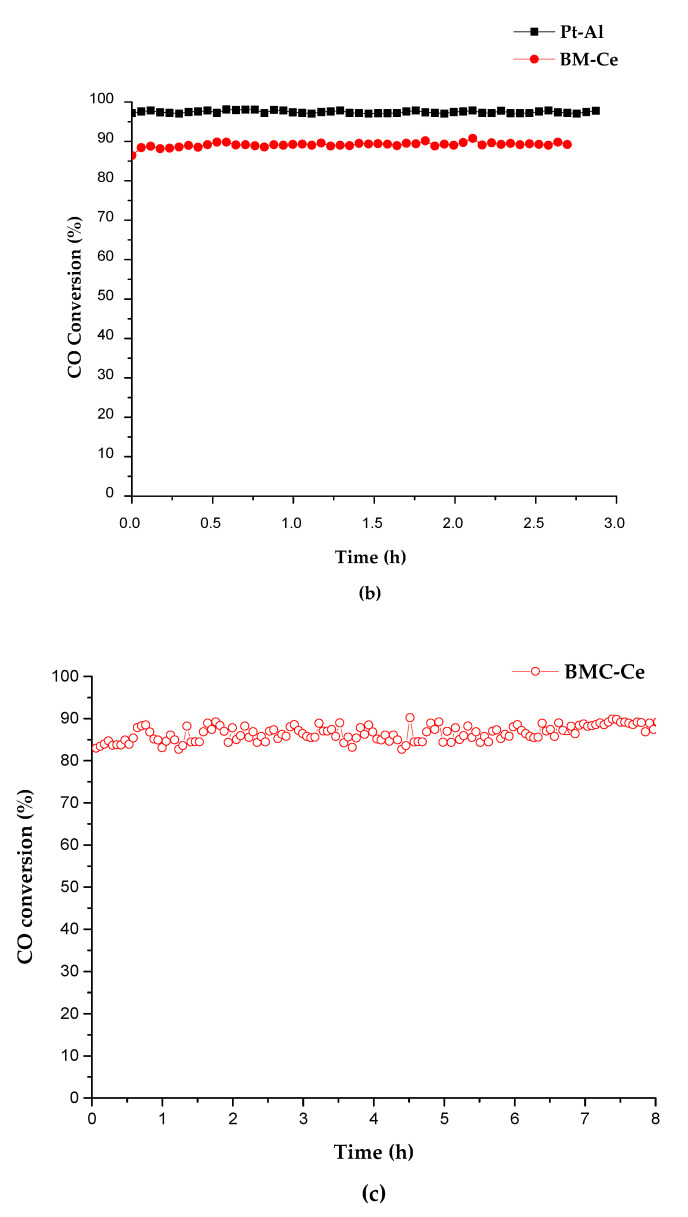
CO conversion profiles at (**a**) 300 °C in 1% CO + 1% O_2_ for all BMC-A samples and Pt-Al, (**b**) 200 °C in 0.1% CO + 10% O_2_ for BMC-Ce and Pt-Al (3 h), and (**c**) 200 °C in 0.1% CO + 10% O_2_ for BMC-Ce (8 h).

To further analyse the performance of BMC-A samples, two cycles (3 h each) of isothermal reactions at 300 °C in the 1% CO and 1% O_2_ gas mixture were carried out, with the CO conversion profiles presented in Figure 6a (where the profile for the Pt-Al reference catalyst is also included). All samples featured almost stable conversion profiles along the reaction time, being also similar for the first and second reaction cycles. Additionally, the CO conversion percentages were close to those achieved during CO-TPR tests (Figure 5), with BMC-La and BMC-Ce showing the highest conversion values. Thus, it was confirmed that the addition of A metal allows for the improvement of the isothermal catalytic performance for the CO oxidation reaction with respect to BMC perovskite, as it was also concluded for BM-A series [21].

On the other hand, by comparing the performance for CO oxidation (at 300 °C and for 1% CO + 1% O_2_ gas mixture) of BMC-A and BM-A series [21], it seems that the effect of Cu was more relevant for Ce than for La and Mg. It was demonstrated by the CO conversion percentages at 300 °C, which were higher for BMC-Ce with respect to BM-Ce (65% versus 50%) than for BMC-La with respect to BM-La (75% versus 65%) or BMC-Mg with respect to BM-Mg (50% versus 40%) [21]. Considering the characterization results, BMC-Ce presented Mn(IV) as the main oxidation state in the bulk (as well as the highest reducibility and the highest oxygen mobility of BMC-A series); meanwhile, BM-Ce presented Mn(III) as the main oxidation state in the bulk. Considering that it is well established that Mn(IV) is more active than Mn(III) for CO oxidation [56,57], the increase in the amount of bulk Mn(IV) in BMC-A with respect to BM-A (caused by the presence of Cu(II)), and only found for BMC-Ce, seems being the main reason for the highest improvement in the catalytic performance featured by BMC-Ce catalyst with respect to BM-Ce.

Finally, considering that BMC-Ce showed the highest CO conversion in the three reactant mixtures tested (see Figure 5 and Table 3), the performance of this catalyst at a temperature lower than 300 °C was analysed. For that, an additional isothermal reaction at 200 °C (3 h) was developed using the most favourable reactant gas mixture composition, that is, 0.1% CO + 10% O_2_. For comparative purpose, the Pt-Al reference was also tested in the same conditions. The conversion profile shown in Figure 6b confirms the high and stable CO conversion percentage achieved using BMC-Ce as catalyst, which was close to that of the reference. In order to confirm the stability of BMC-Ce catalyst, a longer experiment (8 h reaction time) was carried out, with the profile shown in Figure 6c. As no significant deactivation was observed, it seems that BMC-Ce sample is a promising alternative to platinum-based catalysts for CO oxidation in a large excess of oxygen.

## 3. Materials and Methods

### 3.1. Synthesis of Catalysts

Ba_0.9_A_0.1_Mn_0.7_Cu_0.3_O_3_ (A = Ce, La, Mg, denoted as BMC-A) catalysts were obtained by the sol–gel method modified for aqueous medium [58]. The metal precursors used in the synthesis were barium acetate (Ba(CH_3_COO)_2_, Sigma-Aldrich, St. Louis, MO, USA, 99.0% purity); lanthanum nitrate hydrate (La(NO_3_)_3_·H_2_O, Sigma-Aldrich, 99.0% purity); magnesium nitrate hexahydrate (Mg(NO_3_)_2_·6 H_2_O, Sigma-Aldrich, 99.0% purity); cerium(III) nitrate hexahydrate (Ce(NO_3_)_3_·6 H_2_O, Sigma-Aldrich, 99.0% purity); copper(II) nitrate trihydrate (Cu(NO_3_)_2_·3 H_2_O, Panreac, Castellar del Vallès, Spain, 99.0% purity); and manganese(II) nitrate tetrahydrate (Mn(NO_3_)_2_·4 H_2_O, Sigma-Aldrich, 99.0% purity). Additionally, citric acid (C_6_H_8_O_7_, Sigma-Aldrich, 98.5% purity) was added as a complexing agent to prevent the precipitation of metal precursors (using a molar ratio citric acid/Ba of 2). The procedure started by heating a 40 mL solution of citric acid at 60 °C under continuous stirring. Then, the metal precursors were added, and, after, the temperature was increased to 65 °C for 5 h to obtain the gel. A 30% ammonia solution (Panreac) was used to hold the pH at 8.5 along the entire procedure. Finally, the gel was dried at 90 °C for 48 h, and the solid was subsequently calcined at 850 °C for 6 h.

### 3.2. Characterization of Catalysts

For the characterization of samples, the following techniques were used.

The Inductively Coupled Plasma Optical Emission Spectroscopy (ICP-OES) method was used to determine the elemental composition. For the analysis, 10 mg of sample were dissolved in 5 mL aqua regia diluted in 10 mL of distilled water. The analysis was performed in a Perkin-Elmer device model Optimal 4300 DV (Waltham, MA, USA).

An Autosorb-6B device from Quanta Chrome (Anton Paar GmbH, Graz, Austria) was employed to determine the specific surface area by applying the BET method to N_2_ adsorption at −196 °C data. Before the N_2_ adsorption tests, the solids were degassed at 250 °C for 4 h.

The crystalline structure was identified using X-Ray Diffraction (XRD). The X-ray patterns were recorded with a Bruker D8-Advance device, employing the Cu K_α_ radiation (1.4506 Å) and a step rate of 0.4°/min between 20° and 80° 2θ angles.

The composition of surface was determined by X-ray Photoelectron Spectroscopy (XPS) using a Thermo-Scientific K-Alpha photoelectron spectrometer (Thermo Fisher Scientific, Waltham, MA, USA) and an Al K_α_ (1486.7 eV) radiation source. The pressure in the analysis chamber was kept at 5 × 10^−10^ mbar for obtaining the XPS spectra. The binding energy (BE) and kinetic energy (KE) scales were changed by setting the C 1s transition to 284.6 eV, and the BE and KE values were calculated using the spectrometer’s peak-fit software (Thermo Avantage v5.9929).

Temperature-Programmed Reduction with H_2_ (H_2_-TPR) was used to test the reducibility. The tests were developed using 30 mg of sample heated at 10 °C/min from 25 °C to 1000 °C, and a flow of 40 mL/min of a gaseous mixture composed of 5% H_2_/Ar. The experiments were performed in a Pulse Chemisorb 2705 (from Micromeritics, Norcross, GA, USA) equipped with a Thermal Conductivity Detector (TCD) and using a CuO reference sample to quantify the H_2_ consumption.

A Thermal Gravimetric Mass Spectrometry system (TG-MS, Q-600-TA and Thermostar from Balzers Instruments (Pfeiffer Vacuum GmbH, Aßlar, Germany and Balzers, Liechtenstein) were employed to obtain the O_2_–TPD profiles. For these tests, 16 mg of sample were heated at 10 °C/min from room temperature to 950 °C in a 100 mL/min helium gas flow. Before the tests, all samples underwent a 1 h pre-heating process at 150 °C to remove moisture. For following the evolution of H_2_O, CO, O_2_, and CO_2_, the 18, 28, 32, and 44 *m*/*z* signals were registered. A CuO reference sample was also used for the quantification of the amount of oxygen evolved.

### 3.3. Activity Tests

For the CO oxidation tests, three reactant mixtures were employed:

(i) 1% CO and 1% O_2_ in He, as an approximation to the gaseous mixture in the exhaust of a gasoline car engine.
(ii) 1% CO and 10% O_2_ in He, for analysing the effect of using a higher oxygen concentration with respect to (i) conditions.
(iii) 0.1% CO and 10% O_2_ in He, for simulating the CO oxidation in a very large excess of oxygen, which could be close to the CO/O_2_ ratio in the actual Diesel Oxidation Catalytic (DOC) devices or in the exhaust of oxy-fuel engines (excess of O_2_ and very low amount of CO [22]).

The CO oxidation experiments were performed in a U-shaped quartz reactor filled with a mixture of 50 mg of sample and 100 mg of SiC. Two types of experiment were developed using a gas flow of 100 mL/min:

(i) Temperature-Programmed Reaction conditions (CO-TPR) from room temperature to 500 °C and using a heating rate of 10 °C/min.
(ii) Two consecutive reactions at the selected temperature for 3 h.

To clean the catalyst surface, the mixture catalyst-SiC was pre-heated for 1 h at 600 °C in a 5% O_2_/He gas mixture before CO-TPR and before each isothermal reaction cycle.

An Agilent 8860 Gas Chromatograph (Agilent Technologies Spain, Madrid, Spain), equipped with a Thermal Conductivity Detector and two packed columns (Porapack-Q and MolSieve-13X from Agilent Technologies Spain), was used for the quantification of the reaction products. The CO conversion was determined using Equation (1):

(1) $$\frac{CO\;Conversion\;(\%)\;=\;\left({CO}_{in}-{CO}_{out}\right)}{{CO}_{in}}\;\times \;100$$

where CO_out_ is the outlet molar flow rate of CO, and CO_in_ is the inlet molar flow rate.

## 4. Conclusions

Based on the provided characterization and catalytic activity results, the following conclusions can be drawn:

- The partial substitution of the Ba cation by Ce, La, or Mg in BaMn_0.7_Cu_0.3_O_3_ perovskite-type mixed oxides induced a back conversion from the polytype structure of BaMnO_3_ to the hexagonal structure.
- Mn(IV) and Mn(III) coexisted on the surface of all samples. Mn(IV) was the main oxidation state on the surface of all samples, but, in the bulk, it depended on the A metal: Mn(IV) was the main one for BMC-Ce and BMC-Mg, while Mn(III) was for BMC and BMC-La.
- Cu(II) was partially incorporated into the structure of all perovskites.
- The partial substitution of the Ba cation by Ce, La, or Mg cations seemed to increase the mobility of oxygen and the reducibility, with BMC-Ce featuring the highest oxygen mobility and reducibility among the tested samples.
- All Ba_0.9_A_0.1_Mn_0.7_Cu_0.3_O_3_ (A = Ce, La, Mg) perovskite-type mixed oxides were catalytically active for the oxidation of CO under all the reaction conditions tested, being more active in the gaseous mixtures with low CO/O_2_ ratios and showing the highest activity in 0.1% CO and 10% O_2_.
- The addition of A metal increased the catalytic activity for the oxidation of CO at T < 500 °C with respect to BMC. BMC-Ce was the most active catalyst as it combined the presence of surface copper, oxygen vacancies, a high proportion of bulk and surface Mn(IV), and the contribution of the Ce(IV)/Ce(III) redox pair. At 200 °C and using the 0.1% CO + 10% O_2_ gas mixture, the CO conversion achieved using BMC-Ce was very similar to that shown in the presence of a Pt-Al reference catalyst.

## Figures and Tables

**Table 1:** Nomenclature, XRD data, A (Ce, La, or Mg) and Cu metal contents, and BET surface area.

Nomenclature	Molecular Formula	ICP-OES (wt %)		BET (m^2^/g)	Intensity (a.u) ^a^		Average Crystal Size (nm) ^b^	Lattice Strain ^b^	Cell Parameters (Å) ^c^	
		A	Cu							
					Poly	Hex			a	c
BMC	BaMn_0.7_Cu_0.3_O_3_	-	8.0	3	2448	-	30.7	4.5 ×10 ^−3^	5.8	4.3
BMC-Ce	Ba_0.9_Ce_0.1_Mn_0.7_ Cu_0.3_O_3_	2.1	9.2	6	1441	1077	22.4	1.7 × 10^−3^	5.6	4.3
BMC-La	Ba_0.9_La_0.1_Mn_0.7_Cu_0.3_O_3_	5.4	9.8	7	2064	1067	18.6	0.9 × 10^−3^	5.8	4.2
BMC-Mg	Ba_0.9_Mg_0.1_Mn_0.7_Cu_0.3_O_3_	1.0	9.6	3	1246	1100	25.9	2.1 × 10^−3^	5.8	4.3

^a^ Corresponding to the main diffraction peak of BaMnO_3_ polytype structure (Poly) or BaMnO_3_ hexagonal structure (Hex). ^b^ Calculated using the Williamson–Hall method [23]. ^c^ Calculated using the main diffraction peak of BaMnO_3_ polytype structure.

**Table 2:** XPS data.

Catalyst	BEmax Cu(II)_s_ ^a^ (eV)	BEmax Cu(II)_w_ ^b^ (eV)	BEmax Mn(III) (eV)	BEmax Mn(IV) (eV)	BEmax O_L_ (eV)	BEmax O_ads_ (eV)	$\frac{Mn(IV)}{Mn(III)}$	$\frac{Cu}{M}$ (Nominal = 0.15) ^c^	$\frac{O_{L}}{M}$ (Nominal = 1.5) ^c^
BMC	933.1	934.5	641.3	642.4	528.9	530.6	1.2	0.09	0.8
BMC-Ce	933.1	934.8	641.3	642.4	528.8	530.5	1.0	0.07	0.9
BMC-La	933.2	935.1	641.3	642.3	528.9	530.7	1.1	0.10	0.9
BMC-Mg	933.1	935.0	641.1	642.2	528.7	530.9	1.2	0.09	0.9

^a^ s = strong, ^b^ w = weak, ^c^ M = Ba + A+ Mn + Cu.

**Table 3:** T_50%_ and ΔT_50%_ (°C) values under the three reaction conditions tested.

Catalyst	1% CO + 1% O_2_		1% CO + 10% O_2_		0.1% CO + 10% O_2_	
	T_50%_	ΔT_50%_ *	T_50%_	ΔT_50%_ **	T_50%_	ΔT_50%_ ***
BMC	425	---	445	20	315	130
BMC-Ce	260	165	250	10	120	130
BMC-La	300	125	280	20	205	75
BMC-Mg	330	95	300	30	225	75
Pt-Al	265	160	210	55	110	100

* Temperature drop with respect to T_50%_ of BM, ** temperature drop with respect to T_50%_ in 1% CO + 1% O_2_, *** temperature drop with respect to T_50%_ in 1% CO + 10% O_2_.

## Data Availability

Data are contained within the article.
